# Potential therapeutic effect of Qingwen Baidu Decoction against Corona Virus Disease 2019: a mini review

**DOI:** 10.1186/s13020-020-00332-y

**Published:** 2020-05-19

**Authors:** Jianxia Wen, Ruilin Wang, Honghong Liu, Yuling Tong, Shizhang Wei, Xuelin Zhou, Haotian Li, Manyi Jing, Min Wang, Yanling Zhao

**Affiliations:** 1 Department of Pharmacy, Fifth Medical Center of PLA General Hospital, Beijing, China; 2 School of Pharmacy, Chengdu University of Traditional Chinese Medicine, Chengdu, China; 3 China Military Institute of Chinese Medicine, Fifth Medical Center of PLA General Hospital, Beijing, China; 4 Department of Pharmacology, School of Basic Medical Sciences, Capital Medical University, Beijing, China

**Keywords:** Qinwen Baidu Decoction, Corona Virus Disease 2019, Theoretical basis, Functional characteristics

## Abstract

The Corona Virus Disease 2019 (COVID-19) is an acute respiratory infectious disease. At present, COVID-19 has no specific therapeutic drugs, and the main clinical treatment is symptomatic treatment and control of complications. On March 5, 2020, the National Health Commission of the People’s Republic of China issued the *Guidelines for the Diagnosis and Treatment of Novel Coronavirus* (*2019*-*nCoV*) *Infection* (*Trial Version 7*), which integrated traditional Chinese medicine (TCM) into the treatment of COVID-19. The purpose of this study is to summarize recent studies on the clinic application, pharmacological action, chemical substances and mechanism of Qingwen Baidu Decoction (QBD) on the treatment of various diseases. The results suggested that QBD has multiple pharmacological effects such as anti-inflammation, antiviral, antibacterial, immunomodulatory, antipyretic and so on. It has been used in the treatment of sepsis, epidemic hemorrhagic fever, epidemic cerebrospinal meningitis, infantile pneumonia, sepsis-related encephalopathy, epidemic encephalitis B and other diseases. In addition, this study attempts to explore the possible mechanism of QBD in the prevention and treatment of COVID-19. Through the analysis of the chemical substances, pharmacological action and mechanism of QBD, this paper will provide a reference theoretical basis for the prevention and treatment of COVID-19 by QBD.

## Background

Novel Coronavirus Pneumonia (NCP) is an acute infectious pneumonia whose pathogen is novel coronavirus, 2019-nCoV, which has not been previously found in humans. On February 11, 2020, the World Health Organization (WHO) named it as 2019 coronavirus disease, and its English name was Corona Virus Disease 2019 (COVID-19). Since December 2019, a number of pneumonia patients with COVID-19 infection have been found in Wuhan, Hubei Province, China. With the spread of the epidemic, such cases have also been found in other parts of China and worldwide. At present, most of the reported cases have a history of residence or travel in Wuhan, and no case of travel history in Wuhan has been found in some areas. NCP is officially declared a public health emergencies of international concern (PHEIC) on January 30, 2020 by WHO. NCP has been included in the Class B infectious diseases stipulated in the *Law of the People’s Republic of China on Prevention and Control of Infectious Diseases*. Simultaneously, prevention and control measures for class A infectious diseases are taken and implemented. In China, as of 10 AM on May 3, 2020, a total of 84,393 cases have been confirmed, 78,939 cases have been cured and 4643 cases have died, with a mortality rate of 5.50%. NCP has spread all over the world and posed a major threat to human health. There is no vaccine or specific effective drugs for COVID-19 infected pneumonia at present. On March 5, 2020, the National Health Commission of the People’s Republic of China issued the *Guidelines for the Diagnosis and Treatment of Novel Coronavirus* (*2019*-*nCoV*) *Infection* (*Trial Version 7*), which integrated traditional Chinese medicine (TCM) into the treatment of COVID-19. The therapeutic schedule is mainly isolation treatment and symptomatic support treatment.

Traditional Chinese medicine is a great treasure house of China, and it has unique advantages in the treatment of viral infectious diseases. COVID-19, which belongs to the category of “pestilence” and “epidemic disease” in TCM, has the pathogenesis characteristics of “dampness, poison, blood stasis and closure”, and presents the syndrome of “dampness poisoning heat and poisoning lung collaterals” in the progressive stage of pneumonia [1]. The theory of syndrome differentiation characterized by defensive energy nutrients and blood shows its unique advantages in guiding the clinical prevention and treatment of viral infectious diseases with unknown characteristics and strong infectivity. It provides an effective treatment scheme for infectious diseases such as SARS, H1N1, H7N9, and has been internationally recognized [2–4]. Viral infectious disease is called epidemic disease in TCM. It is an acute febrile disease caused by pathogenic *qi* disease invading the human body through mouth, nose or muscle surface. It belongs to the category of febrile disease. TCM emphasizes the overall concept, syndrome differentiation and treatment, which can not only be treated, but also can be prevented, especially in the improvement of body symptoms, which has unique advantages. In the novel coronavirus campaign, TCM is playing a new therapeutic role in different stages of NCP, that is, “supporting the healthy and eliminating the evil, and keeping the same strain”, “syndrome differentiation and treatment to change strain”, restraining “cytokine storm”, and controlling the serious development of the disease, reducing the sequelae caused by hormones and other drugs, decreasing the fatality rate of patients, and so on. The strategy of “TCM is added to the main battlefield of anti-epidemic” is implemented, and the clinical curative effect of the existing Chinese medicine preparation or decoction is observed. Currently, the conventional treatment plus TCM plan has been integrated and published, forming a unified, highly directive and practical “integrative medicine for the prevention and treatment of NCP”.

At present, COVID-19 serves as the pathogenic mechanism of NCP has not been fully revealed. Inhibiting virus proliferation in host cells and reducing host system response is considered to be an important way to alleviate viral infectious diseases. As a β-coronavirus, COVID-19 has more than 85% homology with SARS-like coronavirus (bat-SL-CoVZC45). Angiotensin converting enzyme 2 (ACE2) and coronavirus 3CL Mpro on host epithelial cells affected by its S-protein are considered to be the core targets for inhibiting virus proliferation [5]. Simultaneously, cytokine storm induced by virus is the main cause of inflammation, septic shock and multiple organ failure [6].

In this research, we summarized the pharmacological action, mechanism of action and clinical application of Qingwen Baidu Decoction (QBD), and analyzed its characteristics and advantages of clinical application. Thus, this study will provide theoretical basis and practical reference for its clinical application in anti-NCP and other diseases.

## Brief introduction of QBD

Qingwen Baidu Decoction, which originated from *A View of Epidemic Febrile Diseases with Rashes* (*Yi Zhen Yi De named in Chinese*) in the Qing Dynasty [7], was created by Shiyu Yu, a famous febrile pathologist in the Qing Dynasty. The original prescription of QBD was made from Baihu Decoction, Antidotal Decoction of Coptis and Decoction of Rhinoceros Horn and Rehmannia, including Gypsum Fibrosum, Rehmanniae Radix, Rhinoceros nicornis LR.simus Burchell, Gardeniae Fructus, Platycodonis Radix, Coptidis Rhizoma, Scutellariae Radix, Anemarrhenae Rhizoma, Paeoniae Radix Rubra, Scrophulariae Radix, Forsythiae Fructus, Lophatheri Herba, Glycyrrhizae Radix et Rhizoma, and Moutan Cortex, which has the effect of clearing heat and cooling blood, alleviating heat-producing and disintoxicating. It is one of the most representative anti-epidemic agents in TCM [8, 9]. Clinical evidence suggests that QBD can be used in the treatment of a variety of viral infectious diseases, which can relieve symptoms with a good clinical effect. Pharmacological studies show that the antiviral effect of QBD is mainly associated with Gardeniae Fructus, Coptidis Rhizoma, Scutellariae Radix, and Scrophulariae Radix, which have obvious inhibitory effect on many kinds of influenza viruses via repressing virus replication and preventing virus from entering cells. At the same time, Rehmanniae Radix and Anemarrhenae Rhizoma can significantly regulate cellular and humoral immune function, enhance the activity of immune system and reduce the expression of inflammatory factors [10].

## Clinical research progress of QBD against virus infection related diseases

The pharmacological effect of QBD is exerted through multi-pathway, multi-target and multi-action mechanism, and its modern clinical research is mainly used in the follow aspects.

### Sepsis

Clinically, patients with high fever accompanied by extreme thirst, headache, dysphoria, delirium, red tongue and scorched lips, it is syndrome of flaring heat in *qifen* and *xuefen*, which is difficult to treat. It is common in pneumonia, septicemia, sepsis, infectious mononucleosis and so on in modern medicine. Among them, sepsis is a life-threatening organ dysfunction syndrome caused by the body’s dysfunctional response to infection [11–13]. QBD has effects on relieving sepsis fever, inhibiting early inflammatory reaction, improving blood coagulation dysfunction, and reducing organ function damage, also effectively improving patients’ clinical symptoms, ameliorating patients’ prognosis and so on [14–16]. Nowadays, extensive studies have shown that QBD combined with conventional therapy in the treatment of sepsis can effectively ameliorate the clinical symptoms of patients, reduce or inhibit systemic inflammatory reaction, protect the function of various organs and systems, and improve the prognosis of patients. Conventional therapy combined with QBD in the treatment of sepsis (syndrome of flaring heat in *qifen* and *xuefen*) has effects on reducing the serum immunological indexes such as IgG, IgA, IgM, C3, CRP and TNF-α, reducing Acute Physiology and Chronic Health Evaluation II (APACHE II) score, as well as improving the effective rate of clinical treatment with good clinical effect, which is worth popularizing in clinical [17, 18].

A prospective randomized controlled study was used to observe and evaluate the clinical effect of QBD on blood coagulation indexes and TCM clinical symptom scores in patients with sepsis with blood coagulation dysfunction. The results indicate that QBD can increase the total clinical effective rate of patients with sepsis coagulation dysfunction from 74.2% of the basic treatment to 93.9%. Also, QBD can also significantly decrease the TCM clinical symptom score, APACHE II score and sequential (sepsis-related) organ failure assessment (SOFA). These results indicate that QBD combined with routine treatment can significantly improve the clinical symptoms and blood coagulation function indexes of sepsis patients, ameliorate patient’s health status and protect internal organs [19]. Xi et al. [20] used QBD plus routine treatment to investigate its effect on patients with sepsis with pathogen invading lung defense syndrome. The APACHE II score, C-reactive protein (CRP), white blood cell (WBC) count, TCM syndrome score, blood glucose and antipyretic time were significantly improved in the integrated group, and there was no obvious manifestation of organ failure. It shows that QBD can significantly improve the clinical symptoms and shorten the course of disease. Lin et al. [21] established sepsis model in rats by Cecal ligation and puncture (CLP). It was found that the mechanism of QBD in treating sepsis was related to regulating the expression of genes related to IL-17 signal pathway in rat spleen. Yi et al. [22] established sepsis model by intravenous injection of colibacillus endotoxin into rabbit ear vein. It was found that QBD reduced systemic inflammation in septic rabbits by regulating TLR4/NF-κB signalling pathway. Wang et al. [23] found that the physiological and pathological conditions of septic acute lung injury (ALI) in QBD rats were significantly better than those of the model group (including pathological injury, pathological score, respiratory function, mental state and mortality, etc.), and the expressions of JAK2, p-JAK2, STAT3, p-STAT3, IKKα and NF-κBp65 protein in lung tissue of rats in QBD group were significantly down-regulated. It is suggested that QBD can exert its protective effect by inhibiting the continuous activation of JAK2/STAT3 and IKKα/NF-κB pathway, and its down-regulating effect is significantly related to the improvement of physiological and pathological conditions in rats with ALI. In addition, it can effectively inhibit the lung tissue inflammatory factors (TNF-α, IL-1β, IL-6) and its mediated JAK2/STAT3 signal pathway in septic ALI rats. In addition, QBD could improve the negative feedback regulation mechanism of this pathway, and have a protective effect on ALI model rats [24]. The effects of QBD on lung pathology and the expression of nuclear factor-κBp65 (NF-κBp65) in rats with ALI induced by lipopolysaccharide (LPS) were observed to explore the therapeutic effect and intervention mechanism of QBD on ALI. The results showed that QBD could reduce the degree of lung injury induced by LPS in ALI rats, the accumulation, infiltration and exudation of inflammatory cells in the lungs to a certain extent, playing a definite role in lung protection. QBD can effectively reduce the expression of NF-κBp65 in the lung tissue of ALI rats induced by LPS. It could reduce the “cascade” reaction of inflammation by inhibiting the activation of NF-κB and the production of inflammatory cytokines, as well as inhibiting the inflammatory reaction. The use of QBD in the early stage of ALI induced by LPS can effectively reduce the injury course of lung tissue and the inflammatory reaction, as well as preventing the occurrence and development of inflammation [25].

### Epidemic hemorrhagic fever

Li et al. [26] investigated the potential mechanism of QBD in treating the fever period of hemorrhagic fever with renal syndrome (HFRS). The results showed that QBD combined with conventional therapy was significantly superior to routine therapy in the treatment of HFRS and the former shows its effects on the regulation of cell immune function and the improvement of the HFRS symptoms. By detecting the level of CD^4+^ and CD^8+^ cells, CD^4+^/CD^8+^ ratio and the concentration of TNF-α and IL-10 in lymphocyte, the authors found that the prescription could significantly change the clinical symptoms, and the integrated methods was better than that conventional therapy used alone. According to the different stages of epidemic hemorrhagic fever patients. According to the different stages of epidemic hemorrhagic fever patients, Hao et al. [27] combined conventional therapy with the application of QBD for treatment, the results show that the therapeutic effect of integrated strategy is significant with no adverse reactions.

### Epidemic cerebrospinal meningitis

Qin et al. [28] treated four cases of meningococcal meningitis mainly by oral QBD supplemented by antibiotics. All of them were cured and the course of treatment was shortened. Sun et al. [29] treated 62 patients with epidemic cerebrospinal meningitis with QBD. The results showed that 58 cases were cured, 3 cases were significantly improved and 1 case was ineffective.

### Infantile pneumonia

On the basis of routine treatment, QBD plus subtraction retention enema increased the cure rate of infantile pneumonia from 81.4 to 97.62%, which significantly improved the clinical effect of the patients [30].

### Sepsis-associated encephalopathy (SAE)

Through the intervention of QBD, the relationship between TLR4, cytokines and inflammatory mediators in SAE rats induced by CLP was observed. The results showed that QBD could inhibit the number of WBC, the percentage of neutrophils (NE), TNF-α, IL-6, leukotriene B4 (LTB4) and the content of TLR4 positive cells in brain tissue of rats with sepsis-related encephalopathy. QBD can reduce the excessive inflammatory response and regulate the immune response in sepsis-related encephalopathy rats through its anti-inflammatory factors (TNF-α, IL-6 and TLB4) and regulating the expression of TLR4-mediated inflammatory signal pathway, indicating that QBD can improve the neurological function and inflammatory response of sepsis-related encephalopathy rats [31].

### Epidemic encephalitis B

Hong [32] found that QBD plus Rhei Radix et Rhizoma combined with conventional therapy had obvious advantages over conventional therapy used alone in the treatment of epidemic encephalitis B in children. Yang et al. [33] treated 16 cases of epidemic encephalitis B with oral QBD. The results showed that the effect of QBD was good and the total effective rate was 87.5%.

## Pharmacological research progress of QBD on anti-virus and infection related inflammation

QBD has a wide range of biological activities. Studies have shown that it has the effects of anti-inflammation, anti-virus, antibacterial, protecting liver and gallbladder, regulating immunity, anti-cardiovascular disease and so on. Modern pharmacological studies show that QBD has multiple pharmacological effects, such as antipyretic, reducing blood viscosity, antagonizing platelet aggregation, antibacterial, anti-viral, anti-inflammatory, sedative, analgesic, liver protection, cardiotonic, detoxification, diuresis and so on.

### Anti-inflammatory effect

By dynamically observing the effect of QBD on the expression of serum cytokines TNF-α, IL-8 and IL-10 in rats with ALI induced by non-invasive instillation of endotoxin (LPS, 2 mg/kg) solution through larynx, He et al. [34] found that QBD could regulate the imbalance of pro-inflammatory and anti-inflammatory factors during ALI, alleviate pulmonary inflammatory injury, and thus protect lung tissue. Wang et al. [35, 36] also found that QBD can effectively regulate the expression levels of inflammatory cytokines IL-1β and anti-inflammatory cytokines IL-13 in blood of rats with ALI induced by LPS, promote the dynamic balance of inflammatory and anti-inflammatory cytokines, reduce the total number of white blood cells in bronchoalveolar lavage fluid, improve alveolar injury, inflammatory cell infiltration and erythrocyte exudation, etc., thereby reducing inflammatory cell infiltration in the lung. It can repair and protect the injured lung tissue. Oral or nasal feeding with QBD on the basis of conventional treatment can reduce the expression levels of TNF-α, IL-1, IL-6 and IL-10 in patients with septic acute renal injury, inhibiting excessive inflammation and delaying renal injury [37].

Wang et al. [35] observed the effect of QBD on the level of serum inflammatory factors in rats with ALI, and found that it can effectively regulate the expression of inflammatory cytokines IL-1β and IL-13 to achieve a dynamic balance between inflammatory and anti-inflammatory cytokines, repair and protect the injured lung tissue. Wu et al. [38] observed the anti-inflammatory effects of paeonol and geniposide by increasing celiac capillary permeability and foot swelling test in mice. The results showed that both paeonol and geniposide had obvious anti-inflammatory effects. These results indicate that the anti-inflammatory effect of QBD is closely related to the anti-inflammatory effect of Moutan Cortex and Gardeniae Fructus in the prescription.

### Antiviral effect

Shi et al. [39] used Gardeniae Fructus extract for detecting the expression of VP16 mRNA and IFN-γ in the brain of mice infected with herpes simplex virus type I in vivo. They found that Gardeniae Fructus extract could inhibit the replication of herpes simplex virus in the brain of mice. Therefore, it can be known that QBD has significant antiviral effect, which is related to the large doses of Forsythiae Fructus, Scutellariae Radix, and Gardeniae Fructus and so on. By observing the efficacy of QBD in the treatment of H1N1 influenza virus pneumonia, the results showed that the total effective rate of QBD treatment group (96.0%) was significantly higher than that of the control group (70.0%). And the serum levels of TNF-α, IL-6, IL-8 and CRP in the QBD were lower than those in the control group, while the level of IL-10 was higher than that in the control group. It was effective in the treatment of H1N1 influenza A complicated with respiratory distress syndrome. It shows that QBD can reduce the inflammatory reaction and improve the symptoms of patients, which is of great significance in the prevention and treatment of influenza A H1N1 viral pneumonia. TCM not only has the effect of multi-link anti-influenza A virus, but also can adjust the immune state of the body, improve the antiviral ability, enhance the stability of the tissue itself, reduce the excessive inflammatory reaction of the body, and protect the cell tissue. QBD plays a significant role in reducing fever, reducing temperature, relieving cough and removing phlegm, improving symptoms and other effects, and its curative effect is easy for patients to accept, which reflects the advantages of TCM compound prescription in the treatment of multi-pathway, multi-link and multi-target, and is of great significance to effectively control the epidemic situation of influenza A H1N1 [15, 40].

The clinical observation of QBD combined with ganciclovir in the treatment of infectious mononucleosis (IM) caused by epstein–barr virus infection showed that the total effective rate of the combined group was 93.48%, significantly higher than that of the control group (80.00%), and the effect was more obvious [41]. The curative effect was more obvious and the time of fever, pharyngitis and enlargement of liver and spleen lymph nodes in the QBD group was significantly shortened. The time for the total number of WBC, abnormal lymphocytes and liver function to return to normal was significantly shortened, indicating that QBD is effective in the treatment of infectious mononucleosis in children [42].

To study the clinical efficacy of colon infusion of QBD in the treatment of severe entero virus 71 (EV71) infection. The control group was treated with routine therapy, and the experimental group was treated with routine therapy combined with colon drip of QBD. The number of advanced cases in the experimental group was significantly reduced, and the time of fever regression and hospitalization in the experimental group was significantly shorter than that in the control group. The results show that QBD colonic drip is effective in the treatment of EV71 infection. It is worth popularizing for early antipyretic and preventing disease progression [43].

Shen et al. [44] infected mice by adenovirus ADV3 and influenza virus FM1. Then, mice were treated with different concentrations of Scutellariae Radix and Forsythiae Fructus water extract. It was found that both of them could significantly reduce the mortality of mice infected with the two viruses, indicating that their antiviral effect was significant. The effect of Gardeniae Fructus should not be ignored though served as an adjuvant in this prescription.

In addition, QBD has the effects of antipyretic, anti-inflammatory, antibacterial and antiviral. It can shorten the antipyretic time in the treatment of SARS, and no adverse reactions are found, so it can be used as an antiviral adjuvant in clinic [45].

### Antimicrobial effect

Yu [46] used Coptidis Rhizoma to treat the skin model of bacterial and fungal infection in Japanese white rabbits, and the results showed that it had a strong antibacterial effect. Zhu et al. [47] found that Coptidis Rhizoma and its different processed products had obvious bacteriostatic effect. Han et al. [48] used the different extracts of Anemarrhenae Rhizoma to test the bacteriostasis of *Staphylococcus aureus*, *Shigella dysenteriae*, *Pseudomonas aeruginosa* and *Escherichia coli* in vitro. The results showed that sarsasapogenin in the extract had the strongest antibacterial activity. The Coptidis Rhizoma also has significant antibacterial activity in vitro. Liu et al. [49] used QBD water extract to inhibit *Escherichia coli* and *Klebsiella pneumoniae* in vitro. The results showed that it had inhibitory effect on two kinds of enzyme-producing bacteria. Therefore, it is speculated that the antibacterial effect of QBD may be exerted through the combination of the above drugs.

### Immunomodulatory effect

Using QBD to treat rats with summer heat syndrome of febrile disease, the levels of IL-2, IL-6 and IL-18 in the large dose QBD group and the IL-10 level in the low dose QBD group were higher than those in the LPS control group, and the levels of IL-2 and IL-18 in the high dose QBD were higher than those in the low dose QBD group. QBD can increase the levels of IL-2, IL-6, IL-10 and IL-18 in the rat model of heat–heat syndrome of febrile disease, which may have the effect of immune enhancement [50]. Zhang et al. [51] observed the changes of IgG, IgA, IgM, CRP, TNF-α and C3 in peripheral venous blood of patients with sepsis before and after treatment with QBD. The results showed that QBD could inhibit the excessive immune response of patients and reduce its damage to the body. Moutan Cortex is reused in QBD, and its main active ingredient paeonol has the effect of regulating immunity. Adding QBD on the basis of routine treatment of western medicine can improve the clinical curative effect of patients with sepsis from 75.00 to 84.00%, reduce the symptom score of TCM and the levels of IgG, IgA, IgM, C3, CRP and TNF-α, inhibit the excessive immune response of septic patients and reduce the damage of excessive immune response to the body [52].

Zhang et al. [53] through intraperitoneal injection of 5% chicken erythrocyte suspension and intragastric administration of paeonol of different concentrations. Finally, they found that paeonol could enhance the specific humoral and cellular immune function of mice. Fu et al. [54] treated 18 patients with sepsis by QBD, and detected the peripheral blood prothrombin time, activated partial thromboplastin time, thrombin time and platelet count before and after treatment. The results showed that the prescription can improve the blood coagulation function of patients with sepsis and play a protective role in patients with sepsis.

### Antipyretic effect

Wang et al. [55] observed the clinical efficacy of QBD in the treatment of high fever, and found that the total effective rate of QBD in the treatment of high fever (92.3%) was significantly higher than that in the control group of Angong Niuhuang Pill (57.69%). The clinical effect was satisfactory. Wang et al. [56] studied the changes of body temperature in the fever model of rats and mice induced by dry yeast, endotoxin and 2,4-dinitrophenol, and found that Yuanshen can reduce the fever temperature of rats and mice within 4–8 h after the fever. It is inferred that the antipyretic effect of QBD may be played by Gypsum Fibrosum, Scutellariae Radix, Forsythiae Fructus and so on. Li et al. [57] induced fever in rats by subcutaneous injection of yeast solution, intragastric administration of different doses of baicalin solvent, detected body temperature in different periods of time, and found that its antipyretic effect was significant. Yu et al. [58] induced systemic inflammatory response syndrome in rabbit by injecting LPS into ear vein. After intragastric administration of different doses of Gypsum Fibrosum, the anal temperature was measured and it was found that QBD had obvious antipyretic effect. Hu et al. [59] used different extracts of Fructus Forsythiae shell and seed to observe the neutralization effect on fever of rabbit, ear swelling of mouse and endotoxin. The results showed that the antipyretic effect of the extracts was obvious.

## Material basis of anti-virus and infection-related inflammation of QBD

In QBD, the sovereign drug is Gypsum Fibrosum, and its main active ingredient is hydrous calcium sulfate; Rehmanniae Radix is a minister drug, which mainly contains rehmannia glycoside, catalpol and so on; The adjuvants are Coptidis Rhizoma, Scutellariae Radix, Anemarrhenae Rhizoma, Scrophulariae Radix, Gardeniae Fructus etc. Coptidis Rhizoma mainly contains berberine. Scutellariae Radix contains a lot of baicalin. the main active ingredients of Anemarrhenae Rhizoma are sarsasapogenin. Scrophulariae Radix contains paeonol. Gardeniae Fructu mainly contains geniposide and geniposide. Paeoniae Radix Rubra mainly contains paeoniflorin, etc. The main components of Forsythiae Fructus are phillyrin and forsythoside, Rhinoceros nicornis LR.simus Burchell mainly contains cholesterol and so on. Pentacyclic triterpenoid glycosides are the main active components of Platycodonis Radix, and the effective components of Moutan Cortex are paeonol and paeoniflorin. The conductant drug of this prescription are fresh Lophatheri Herba and Glycyrrhizae Radix et Rhizoma, which contain flavonoids, glycyrrhizin and glycyrrhetinic acid, respectively [60].

Gypsum Fibrosum and Rhinoceros nicornis LR.simus Burchell powder are commonly used and effective antipyretic in TCM. Scutellariae Radix, Forsythiae Fructus, Scrophulariae Radix and Gardeniae Fructus can not only assist the main drug in heat-clearing and detoxification, but also have direct antiviral effects confirmed by modern pharmacological studies. TCM believes that “superheat consuming qi” and “consumption of yin caused by febrile diseases”. Thus, both heat-clearing and detoxification and replenishing qi and nourishing yin should be taken into accounts in treatment. Astragali Radix, Radix et Rhizoma, Anemarrhenae Rhizoma, and Rehmanniae Radix were added to the original prescription, while Astragali Radix can achieve indirect antiviral effect by regulating the immune function of the body [45].

Through the analysis of the compatibility of disassembled prescriptions and the parallel comparison of multiple indexes, Ding et al. [61] selected the effective ingredient of QBD to clear away heat and purge fire, and made it clear that the effective component of QBD in interfering with endotoxic ALI in rats was crocin-1.

## The pharmacological effect of QBD in animal and clinical study

Studies have shown that LPS or endotoxin were mainly used to establish ALI model of rats. Moreover, endotoxin of *Escherichia coli* was used to establish sepsis models in New Zealand rabbit. These studies mainly investigated the influence of QBD on ALI, sepsis or SAE model in terms of lung histopathology, W/D ratio, WBC level, PaO2 level, PaCO2 level, and other indicators. The specific details of disease types, animal types, experimental model, does of QBD, medical effects in QBD group and targets are shown in Table 1. Notably, the relevant research of QBD is mainly focused on clinical research. In clinical, the therapeutic effects of QBD on sepsis, viral pneumonia caused by influenza A virus subtype H1N1, SARS, hyperthermia and other diseases were discussed by collecting clinical cases. In general, QBD combined with conventional therapy can significantly improve the clinical treatment efficiency of these diseases. The reported indicators in QBD group are shown in Table 2.

**Table 1:** The pharmacological effect of QBD in animal experimental study

Disease	Animals	Experimental model	Doses (doses, route)	Pharmacological effects in QBD group	Targets	References
ALI	Male SD rats, weighting 200–250 g	LPS (2 mg/kg, intratracheal instillation)	QBD (7.395 g/kg, 14.79 g/kg, 29.58 g/kg, i.g.)	(1) WBC↓ (2) Lung tissue damage and inflammatory infiltration was improved (3) W/D ratio of lung↓	IL-8↓, TNF-α↓, IL-10↑	He et al. [34]
ALI	Male SD rats, 200–250 g	LPS (2 mg/kg, intratracheal instillation)	QBD (7.395 g/kg, 14.79 g/kg, 29.58 g/kg, i.g.)	Inflammation score of lung histopathology in rats↓	NF-κB↓, p65↓	Wang et al. [25]
ALI	Male SD rats, weighting 200 g	LPS (5 mg/kg, intratracheal instillation)	Ethanol extraction of QBD (i.g.)	The lung volume and bleeding point of rats were significantly reduced. The color of lung tissue is dark or close to pink	NR	Ding et al. [61]
ALI	Male SD rats, 200–220 g	LPS (5 mg/kg, intratracheal instillation)	QBD dismantling (i.g.)	(1) WBC↓, PMNs↓, TNF-α↓ (2) W/D ratio of lung↓, protein concentration↓ (3) Congestion, edema and inflammatory cell infiltration of pulmonary interstitium and alveoli were decreased	NR	Zhang et al. [62]
ALI	Male Wistar rats, 220 ± 20 g	Endotoxin (3 mg/kg, i.v.)	QBD (7.5 g/kg, 15 g/kg, i.g.)	(1) Respiratory rate↓ (2) PaO2↑, PaCO2↓ (3) Pathological score↓	JAK2↓, p-JAK2↓, STAT3↓, p-STAT3↓, p38↓, p-p38↓, A-Raf↑, p-A-Raf↓	Wang et al. [63]
ALI	Male SD rats, 200–250 g	LPS (2 mg/kg, intratracheal instillation)	QBD (7.395 g/kg, 14.79 g/kg, 29.58 g/kg, i.g.)	Improvement in lung pathology	IL-1β↓, IL-13↑	Wang et al. [35]
ALI	Male SD rats, 200–250 g	LPS (2 mg/kg, intratracheal instillation)	QBD (7.395 g/kg, 14.79 g/kg, 29.58 g/kg, i.g.)	(1) WBC↓ (2) W/D ratio↓, lung coefficient↓ (3) Pathology inflammation score↓	NR	Wang et al. [36]
ALI	Male Wistar rats, 180–220 g	Endotoxin (3 mg/kg, i.v.)	QBD (7.5 g/kg, 15 g/kg, i.g.)	(1) EVLW↓, PaO2↑, PaCO2↓ (2) Pathological type II alveolar cell injury↓	(1) IL-1β↓, IL-6↓, TNF-α↓ (2) p-JAK2↓, p-STAT3↓, SOCS1↑, PIAS3↓	Wang et al. [24]
Sepsis	New Zealand rabbits, 2.19 ± 0.135 kg	Endotoxin of *Escherichia coli* (10 μg/kg, auricular vein injection)	QBD (873 mg/kg, i.g.)	Net increase in fever↓	TLR4↓, NF-κB↓, IL-6↓, TNF-α↓	Yi et al. [22]
Epidemic febrile disease	Male SD rats, 346 ± 26 g	Lead acetate (25 mg/kg) + LPS (15 mg/kg), caudal vein injection	QBD (15.60 g/kg, 30.29 g/kg, i.g.)	(1) Systolic pressure↑, diastolic pressure↑, mean arterial pressure↑, heart rate↑ (2) Mean time to live estimate↑, median lifetime estimate↑	NR	Yu et al. [64]
SAE	Male SD rats, 250 ± 50 g	CLP	QBD (i.g.)	(1) Neurological score↑ (2) WBC↓, NG↓ (3) TLR4 positive cells↓	TNF-α↓, IL-6↓, LTB4↓	Zheng et al. [31]
Acute peritonitis	Male SD rats, 200 g	CLP	QBD (30.8 g/kg, 40.8 g/kg, i.g.)	NR	IL-8↓, IL-10↑, TNF-α↓	Wang et al. [65]

*QBD* Qingwen Baidu Decoction, *LPS* lipopolysaccharide, *ALI* acute lung injury, *CLP* cecal ligation and perforation, *W/D* wet to dry ratio, *SAE* sepsis-associated encephalopathy, *NG* neutrophil granulocyte, *LTB4* leukotriene B4, *EVLW* extravascular lung water, *NR* not report

**Table 2:** The pharmacological effect of QBD in clinical trials

Disease	Gender (M/F)	Age (years) (range, mean)	Interventions	Course	Clinical efficiency	Reported indicators in QBD group	References
Sepsis	E: 11/7 C: 12/8	E: 53.27 ± 9.24 C: 51.00 ± 11.29	E: Conventional therapy + QBD (100 ml/time, tid.) C: Conventional therapy	7 days	E: 83.33% C:75.00%	IgG↓, IgA↓, IgM↓, C3↓, CRP↑, TNF-α↓, APACHEII score↓, TCM symptom score↓	Zhang et al. [51]
Sepsis	E: 18 C: 20	NR	E: Conventional therapy + QBD (100 ml/time, tid.) C: Conventional therapy	7 days	E: 83.33% C: 75.00%	TCM symptom score↓, APACHEII score↓, IgG↓, IgA↓, IgM↓, C3↓, CRP↑, TNF-α↓	Leng et al. [18]
Sepsis	E: 11/7 C: 12/8	E: 53.27 ± 9.24 C: 51.00 ± 11.29	E: Conventional therapy + QBD (200 ml/time, tid.) C: Conventional therapy	7 days	E: 83.30% C: 75.00%	TCM symptom score↓, APACHEII score↓, mortality rate↓, PT↓, APTT↓, TT↓, PLT↑	Fu et al. [54]
Sepsis	E: 15/10 C: 16/8	E: 53.27 ± 9.20 C: 51.00 ± 11.29	E: Conventional therapy + QBD (100 ml/time, tid.) C: Conventional therapy	7 days	E: 84.00% C: 75.00%	TCM symptom score↓, APACHEII score↓, IgG↓, IgA↓, IgM↓, C3↓, CRP↑, TNF-α↓	Leng et al. [52]
Sepsis	E: 19/23 C: 22/20	E: 50–74, 61 ± 8.6 C: 52–75, 63 ± 7.3	E: Antibiotic therapy + QBD (100 ml/time, bid.) C: Antibiotic therapy	7 days	E: 80.95% C: 64.28%	APACHEII score↓, IgG, ↓ IgA↓, IgM↓, C3↓, CRP↑, TNF-α↓	Chen [17]
AKI with sepsis	E: 28/29 C: 30/26	E: 68.10 ± 9.07 C: 69.49 ± 8.37	E: Conventional therapy + QBD (200 ml/day, bid.) C: Conventional therapy	7 days	NR	APACHEII score↓, Scr↓, BUN↓, CRP↓, TNF-α↓, IL-1β↓, IL-6↓, IL-10↓	Ge et al. 2014 [37]
Pathogen-invading-lung syndrome with sepsis	E: 15/9 C: 13/11	E: 68.10 ± 9.07 C: 69.49 ± 8.37	E: Conventional therapy + QBD (100 ml/time, bid.) C: Conventional therapy	7 days	E: 95.83% C: 83.33%	APACHEII score↓, CRP↓, WBC↓, TCM symptom score↓, blood glucose↑, antipyretic time↓	Xi et al. [20]
Sepsis with heat poisoning syndrome	E: 35/24 C: 37/21	E: 46–68, 50.4 ± 8.5 C: 44–70, 51.8 ± 7.9	E: Conventional therapy + Xuebijing injection (100 mg/time, bid.) + QBD (200 mg/time, bid.) C: Conventional therapy	7 days	E: 77.97% C: 42.37%	APACHEII↓, SOFA score↓, TNF-α↓, IL-1β↓, IL-6↓, IL-10↑, d-lactic acid↓, endotoxin↓, PCT↓, whole blood viscosity↓, plasma viscosity↓, erythrocyte deformation index↓, erythrocyte aggregation index↓, PT↑, APTT↓, TT↑, FIB↓, D–D↓, PLT↓	Ren et al. [14]
Coagulation dysfunction in sepsis	E: 20/13 C: 19/12	E: 28–78, 60.94 ± 12.13 C: 28–79, 61.45 ± 12.91	E: Conventional therapy + QBD (100 ml/day, bid.) C: Conventional therapy	7 days	E: 93.90% C: 74.20%	PLT↑, PT↓, APTT↓, Fib↓, D–D↓, TCM symptom score↓, APACHEII score↓, SOFA score↓	Gao et al. [19]
Influenza A (H1N1) with respiratory distress syndrome	2/11	12–61, 29.4 ± 14.1	Conventional therapy + QBD	7–48 days	92.31%	11 cases were cured, 1 case was effective and 1 case was ineffective	Chu et al. [40]
Viral pneumonia caused by influenza A virus subtype H1N1	E: 28/22 C: 24/16	E: 40.67 ± 2.09 C: 41.68 ± 4.68	E: Conventional therapy + QBD (200 ml/day, tid.) + Tanreqing injection (30 ml/day, qd.) C: Conventional therapy	7 days	E: 96.00% C: 70.00%	TNF-α↓, IL-6↓, IL-8↓, IL-10↑, CRP↓, TCM symptom score↓	Tian et al. [15]
SARS	4/6	15–70, 43.77 ± 12.68	Conventional therapy + QBD (150 ml/time, bid.)	5 days	NR	Antipyretic time↓, temperature gradually drops to normal level	Zhang et al. [45]
EV 71	E: 22/8 C: 21/9	E: 2.0 ± 1.3 C: 2.1 ± 0.25	E: Conventional therapy + QBD (50 ml/time, bid. ~ tid.) C: Conventional therapy	3–5 days	E: 93.33% C: 63.40%	Antipyretic time↓, hospital stay↓	Zhan et al. [43]
Epidemic encephalitis type B	10/6	1–11, 5.3	QBD (tid.)	NR	87.50%	10 cases were cured, 4 cases were improved and 2 cases were ineffective.	Yang [33]
Pneumonia	E: 30 C: 30 (36/24)	E: 20–70, 53.11 ± 10.58 C: 18–67, 49.00 ± 10.61	E: Levofloxacin + QBD (150 ml/time, bid.) C: Levofloxacin (0.5 g, qd.)	7 days	E: 96.67% C: 86.67%	Antipyretic time↓, WBC↓, NG↓	Nie et al. [66]
Mycoplasma pneumonia	E: 20/25 C: 22/23	E: 9–58, 27 C: 10–63, 25	E: Azithromycin (10 mg/kg/day, i.v.) + QBD + Canopy bulk C: Azithromycin (10 mg/kg/day, i.v.)	4 W	E: 100.00% C: 86.67%	Blood routine was normal, clinical symptoms disappeared, chest X-ray improved	Shu [67]
Infant pneumonia	E: 22/20 C: 23/20	E: 0.58–9 C: 0.58–8	E: Conventional therapy + QBD (3–5 ml/kg, bid.) C: Conventional therapy	5 days	E: 97.62% C: 81.40%	NR	Yao et al. [30]
Epidemic hemorrhagic fever	E: 84/36 C: 40/20	E: 10–17 (13 cases); 18–49 (82 cases), 50–70 (25 cases) C: 10–17 (4 cases); 18–49 (47 cases), 50–70 (9 cases)	E: Ribavirin (15 mg/kg, qd.) + QBD (bid.) C: Ribavirin (15 mg/kg, qd.)	NR	NR	Days of fever↓, polyuria↓, platelet recovery↓, urine protein turning negative↓, the rate of oliguria and hypotension shock↑, adverse reactions ↓	Hao et al. [27]
Hyperthermia	E: 14/12 C: 11/15	E: 18–62 C: 19–63	E: QBD (150 ml/time, bid.) C: Angong Niuhuang Pill (1 pill, qd ~ bid.)	NR	E: 92.30% C: 57.69%	NR	Wang et al. [55]
Hemorrhagic fever with renal syndrome	E: 45 C: 45 (51/39)	24–48	E: Conventional therapy + QBD (bid.) C: Conventional therapy	7 days	NR	CD4^+^↑, CD8^+^↓, CD4^+^/CD8^+^↑	Li et al. [26]
Infectious mononucleosis	E: 46 C: 45 (43/48)	0.83–6	E: Ganciclovir (10 mg/kg d, bid.) + QBD (bid. ~ tid.) C: Ganciclovir (10 mg/kg d, bid.)	7 days	E: 93.48% C: 80.00%	NR	Dong [41]
Infectious mononucleosis in children	E: 11/9 C: 12/8	E: 2–13 C: 3–12	E: ganciclovir (5 mg/kg) + QBD (200 ml/time, qid.) C: ganciclovir (5 mg/kg)	7–14 days	E: 95.00% C: 80.00%	Duration of main clinical manifestations↓, WBC↓, heterotypic lymphocyte↓, liver function indices↓	Sheng et al. [42]
Epidemic cerebrospinal meningitis	40/22	8–40, 18	QBD (tid.)	15 days	98.39%	NR	Sun et al. [29]
Hand-foot-and-mouth disease	E: 30/20 C: 25/25	E: 1–3 (37 cases), 4–5 (13 cases) C: 1–3 (36 cases), 4–5 (14 cases)	E: Ribavirin (10–15 ml/kg, qd.) + QBD (100–150 ml/day, tid. ~ qid.) C: Ribavirin (10–15 ml/kg, qd.)	10 days	E: 94.00% C: 88.00%	Antipyretic time↓, disappearance time of oral herpes↓, severe conversion rate↓	Xu et al. [68]
Erythroderma psoriasis	E: 14/15 C: 16/13	E: 20–63, 46.35 ± 2.16 C: 20–64, 47.32 ± 2.61	E: QBD (200 ml/time, tid.) C: Acitretin capsules (20 mg/kg)	3 M	E: 96.55% C: 75.86%	Adverse reactions ↓	Li [69]

*E* experimental group, *C* control group, *W* week, *APACHE* acute physiology and chronic health evaluation, *M* month(s), *WBC* white blood cell, *EV* enterovirus, *SOFA* sequential organ failure assessment, *NG* neutrophil granulocyte, *CRP* C-reactive protein, *PLT* platelet count, *PT* prothrombin time, *APTT* activated partial thromboplastin time, *TT* thrombin time, *Fib* fibrinogen, *D-D* D-Dimer, *AKI* acute kidney injury, *BUN* blood urea nitrogen, *NR* not report

## Effect of QBD on lung histopathology

The effect of QBD on lung histopathology was mainly reported in animal experiments. He et al. [34] found that the alveoli space of LPS induced-ALI rats became narrow with reduced swelling of capillary endothelial cells, and the bleeding and exudation of edema fluid were significantly improved in QBD group. Wang et al. [25] found that QBD can reduce the damage degree of ALI rats’ lung tissue, and can reduce the aggregation, infiltration and exudation of inflammatory cells in the lung tissue, so as to play a more obvious role in protecting the lung tissue. QBD can effectively decrease the relative protein expression of NF-κB p65 in ALI rats induced by LPS. By inhibiting the activation of NF-κB and the production of inflammatory cytokines, the inflammatory response of rat lung tissue can be reduced. Early application of QBD can effectively reduce the degree of lung injury and the inflammatory response of alveoli. Zhang et al. [62] found that QBD reduced the degree of congestion and edema of pulmonary interstitium and alveoli, and decreased the infiltration of inflammatory cells. Wang et al. [24] used endotoxin to establish sepsis ALI. The results indicated that pathological type II alveolar cell injury score of observation data significantly reduced in QBD group. In addition, QBD could significantly alleviate the alveolar pathological damage in endotoxin-induced ALI in rats. The results showed that the alveolar structure of QBD group was relatively complete, and the consolidation of alveoli, infiltration of inflammatory cells and edema in alveoli were alleviated in varying degrees.

## Pharmacological effects in QBD group

ALI is a syndrome caused by severe infection, trauma and shock, which is characterized by diffuse high permeability pulmonary edema and parenchymal cell injury. It is a serious complication in the early stage of sepsis. In essence, the disease is an out of control systemic self-destructive inflammatory response. Previous studies have shown that QBD can reduce WBC, PMNs, W/D ratio, PaO2 and PaCO2 levels of LPS-induced ALI [24, 25, 31, 34–36, 61–63]. It was found that the pharmacodynamics of QBD was mainly related to the regulation of IL-8, IL-10 and TNF-α levels [34]. Pathway study showed that QBD could significantly reduce the over activation of NF-κB p65, JAK2/STAT3 and p38 MAPK signaling pathway [25, 63]. Simultaneously, QBD can decrease the degree of lung inflammation and injury, and the over release of inflammatory factors in ALI rats.

## The pharmacological effect of QBD in clinical trials

QBD can be used in the treatment of sepsis, influenza A H1N1 virus pneumonia, SARS, pneumonia, and erythroderma psoriasis and so on, among which the treatment of sepsis is the most reported (Table 2). Most of the collected clinical cases were treated with conventional therapy combined with QBD. The course of treatment was generally about 7 days, which could significantly improve the clinical efficiency. For the treatment of sepsis, the influence of QBD on the APACHE II score, TCM symptom score, serum immunology index, serum PT, APTT, TT and PLT values, mortality and safety of the patients was focused. As sepsis patients have obvious immune dysfunction, QBD can play a role in treating sepsis by regulating the immunological index, and the clinical effect is better than that of the conventional treatment group. Relevant detection indicators of other diseases are shown in Table 2.

NCP is mainly characterized by pneumonia, fever, cough, fatigue, and gradually dyspnea. A few patients have gastrointestinal symptoms such as nausea, vomiting and diarrhea. Currently, novel coronavirus infection has not been confirmed effective specific antiviral drugs. From the outbreak of SARS in 2003 and MERS in 2018 to the recent outbreak of COVID-19, clinicians and scientists have provided several potentially effective antiviral drugs for clinical use, including remdesivir, lopinavir/ritonavir, convalescent plasma and monoclonal antibodies. However, the specific efficacy and safety of these drugs in patients with COVID-19 still need further clinical trials.

**Fig. 1:**
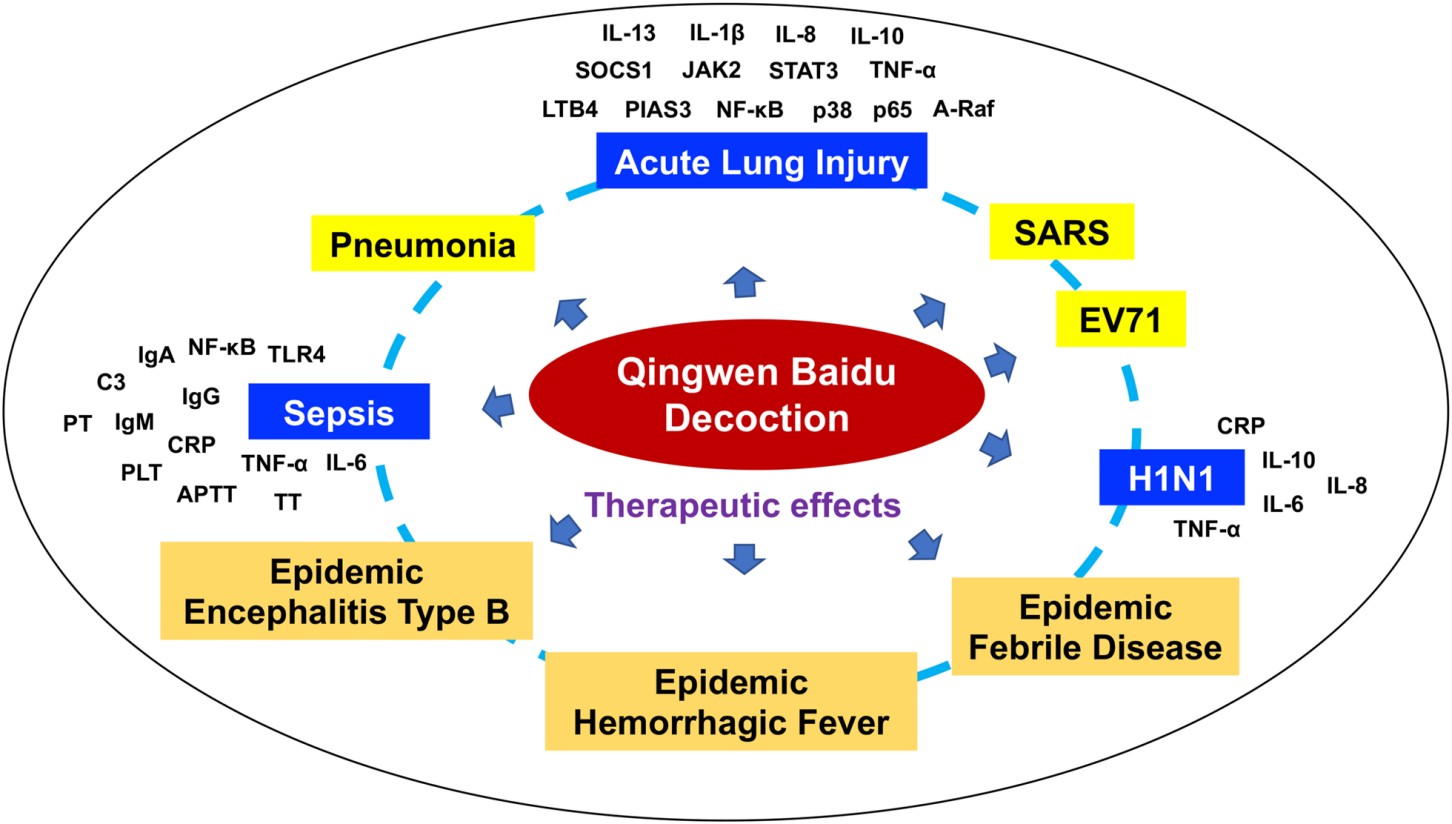
Pharmacological effect and multi-target mechanism of QBD in the treatment of various diseases

Modern pharmacological studies have found that QBD has significant pharmacological activities in antiviral, regulating immune function, inhibiting inflammation, improving vomiting and diarrhea. From the theory of TCM to modern clinical and pharmacological studies, it has been shown that QBD combined with modern medical conventional treatment has therapeutic effect on viral infection related diseases, and its mechanism includes inhibition of virus, infection related inflammation and improvement of disease-related symptoms. QBD has therapeutic effect on a variety of virus (influenza virus H1N1, SARS virus, EV 71, etc.) infection [40, 43, 45]. It is a clinical goal for the body to remove the virus from the body’s immune system. Pharmacological studies show that QBD and its components have significant anti-inflammatory effects, which can inhibit inflammatory exudation, reduce the levels of a variety of pro-inflammatory cytokines, increase the level of anti-inflammatory cytokines IL-10, and regulate the inflammatory related NF-κB/p65, JAK2/STAT3, TLR4 pathways. It is suggested that NCP can alleviate inflammation in QBD, protect organs from inflammation and slow down the progress of inflammation. In addition, QBD could improve the symptoms of fever and diarrhea associated with viral infection, suggesting that QBD may also improve the symptoms of new crown pneumonia. The pharmacological effect and multi-target mechanism of QBD in the treatment of various diseases are shown in Fig. 1.

## Possible mechanism of QBD in prevention and treatment of COVID-19

The inflammation caused by virus infection can promote the clearance of invading virus, and return to normal under negative feedback regulation after virus clearance. However, excessive inflammatory reaction can lead to more serious damage to the body than the virus infection itself, and in some cases even lead to the imbalance of immune regulation, the lack of negative feedback and the continuous amplification of positive feedback, and the abnormal increase of a variety of pro-inflammatory cytokines, resulting in a storm of cytokines, which eventually leads to organ damage, functional failure and even death. COVID-19 patients also experienced a cytokine storm and related acute respiratory distress syndrome (ARDS). In the absence of a specific drug for COVID-19, the clinical goal is to support the treatment and control of inflammation and let the body immune system clear the virus.

The pathogenesis of COVID-19 is not clear. It may be related to the damage of immunity and respiratory epithelial cells caused by COVID-19, and the susceptibility of the body, but regardless of the pathogenesis. It is reported that there is overexpression of inflammatory factors in patients with advanced COVID-19, which leads to cytokine storm [70]. Cytokine storm is considered to be an important factor in the development of viral pneumonia. The roles of cytokines in immune network in promoting disease progression, inducing local inflammation, eliminating infection, regulating cellular and molecular immune response, and regulating tissue repair are very complex [71]. When the immune system is out of balance and cytokines are overexpressed, there will be a cytokine storm, which will cause serious damage to the body, such as diffuse alveolar injury, hyaline membrane formation, fibrin exudation, etc. At the same time, accelerated lung injury, the emergence of more serious pulmonary capillary injury and damage to the immune function of the body, cytokines in the circulatory system will lead to systemic cytokine storm, further lead to systemic organ dysfunction, accompanied by the overexpression of inflammatory factors such as IFN, TNF, IL and so on. Regulating the immune balance in the state of COVID19 and inhibiting the occurrence of cytokine storm will be an important way and mechanism to block the deterioration of the disease.

Overall, QBD has been shown to have a therapeutic effect on exogenous diseases related to viral infection from the theory of TCM to modern clinical and pharmacological studies. QBD and its basic prescription exert the effect of “detoxifying and cooling blood”, and play a significant therapeutic effect on a variety of viral infections (EB virus, H1N1 influenza virus, SARS virus, EV71, etc.). It may be related to the systematic regulation of inhibition of virus proliferation in the host and excessive expression of host inflammatory response, and is expected to be used in the treatment of severe pneumonia in COVID-19. The specific mechanism of its curative effect may be closely related to the inhibition of COVID-19 invasion into the host for proliferation and the improvement of cytokine storm. The replication cycle of animal virus infection has similarity. With the joint efforts of colleagues in the pharmaceutical industry, it has been found that a number of drugs developed for other viruses can inhibit COVID-19 in vitro [72, 73]. Based on the pharmacological effects of QBD, such as antipyretic, antibacterial, antiviral, anti-inflammatory, regulating immunity and so on, it is commonly used in clinical treatment of infectious diseases, such as sepsis, epidemic cerebrospinal meningitis, epidemic hemorrhagic fever, epidemic encephalitis B and so on, with remarkable curative effect, not easy to relapse and little side effect. As far as the material basis of the prescription is concerned, the future research should not be limited to the application of the whole prescription, but should be flexibly applied. The relevant components and contents can be added or decreased according to the disease, and the combination of TCM and western medicine should be adhered.

QBD has a dual regulatory effect on immune function, through the regulation of immunity to achieve the purpose of anti-infection and anti-inflammation [74]. The combination of heat-clearing and detoxifying drugs and drugs for promoting blood circulation and removing blood stasis can enhance the non-specific anti-infective effect of the former [75]. Modern experimental studies have found that QBD has some pharmacological effects, such as heat-clearing and detoxification, anti-platelet aggregation, reducing blood viscosity, anti-inflammation, analgesia, sedation, antibacterial, antiviral, liver protection, cardiotonic, diuretic and so on [76]. All the heat-clearing and detoxifying drugs such as Anemarrhenae Rhizoma, Gypsum Fibrosum, Scrophulariae Radix, Forsythiae Fructus, Coptidis Rhizoma have certain antiviral ability, which can remove the virus or inhibit its replication, and reduce the inflammatory damage of the virus. Forsythiae Fructus can stimulate mononuclear macrophage system, enhance phagocytosis and promote antibody production; Scrophulariae Radix can prolong the existence time of antibody; Scutellariae Radix can enhance leukocyte phagocytosis, promote lymphocyte transformation and improve immune function. Drugs for promoting blood circulation and removing blood stasis such as Paeoniae Radix Rubra and Moutan Cortex can not only inhibit cellular immunity and possibly inhibit the extensive toxic effect of T cells, so as to reduce multiple organ damage, but also promote non-specific immunity, antivirus, regulate immunity and so on [77–82]. Moutan Cortex cool blood and dissipate blood stasis, rash, clear camp diathermy to nourish Yin. Scutellariae Radix, Glycyrrhizae Radix et Rhizoma, Gardeniae Fructus have effect on protecting liver function and forsythia. Forsythiae Fructus, Scutellariae Radix, and Lophatheri Herba can promote the excretion of endotoxin and virus. All kinds of medicines are compatible, clearing heat and detoxification, activating blood circulation and removing blood stasis, replenishing qi and nourishing yin, and by regulating immunity and other effects of antivirus, antipyretic, detoxification and liver protection, so as to achieve the purpose of treatment. It is suggested that QBD can reduce the inflammatory reaction of COVID-19 patients and may have a protective effect on organ damage caused by inflammation. Although there is no complete data on the treatment of COVID-19 by QBD, it is believed that in the near future, the TCM and modern medicine can achieve positive clinical treatment effect and reduce the suffering of patients, improve prognosis and rehabilitation.

In this fight against NCP, TCM has shown its unique advantages in reducing mortality and improving cure rate [83, 84]. Notably, TCM also pays attention to three elements in the treatment of diseases: first, the climate environment. Now, people are paying attention to the relationship between the climate humidity and temperature on the epidemic situation, which is a common problem. Second, we should attach importance to special disease and specific prescription and syndrome differentiation and treatment. Third, we should attach importance to people’s constitution. Although QBD can be used in the treatment of a variety of viral infectious diseases and alleviates the symptoms of the disease with good clinical efficacy, from the perspective of drug properties, QBD belongs to “cold medicine”. If the patient’s normal constitution is partial to *Yang* deficiency, it is not suitable to take more and use for a long time to avoid damaging the organism.

## Conclusions

Currently, the number of convalescent patients of COVID-19 is increasing, especially in clinical practice, by “strengthening the integration of traditional Chinese and modern medicine”, the course of disease is shortened and the cure rate is improved. Modern pharmacology and clinical research also confirmed that the treatment of QBD in COVID-19 has the theory of TCM and modern research results as the theoretical basis, which is suitable for the treatment of patients with COVID-19. It is speculated that its main role is to reduce the level of inflammation in patients. Simultaneously, it plays a role in inhibiting the replication and infection of virus, inhibiting the concurrent bacterial infection, and improving the immunity of the body. However, there are few researches on the mechanism of QBD’s action on human coronavirus. Through the study on the mechanism of QBD’s multi-channel anti coronavirus action, we hope to provide new ideas for the clinical treatment of COVID-19, which improve the cure rate of patients infected with COVID-19, reduce their mortality, and further explore the pharmacological effect and phase of QBD. Thus, the series researches will provide scientific basis for the study and development of new drugs.

## Data Availability

The data used to support the findings of this study are available from the corresponding author upon reasonable request.

## Abbreviations

NCP: Novel Coronavirus Pneumonia
WHO: World Health Organization
COVID-19: Corona Virus Disease 2019
PHEIC: Public health emergencies of international concern
TCM: Traditional Chinese medicine
ACE2: Angiotensin converting enzyme 2
QBD: Qingwen Baidu Decoction
CRP: C-reactive protein
WBC: White blood cell
CLP: Cecal ligation and puncture
ALI: Acute lung injury
LPS: Lipopolysaccharide
HFRS: Hemorrhagic fever with renal syndrome
W/D: Wet to dry ratio
SAE: Sepsis-associated encephalopathy
NG: Neutrophil granulocyte
LTB4: Leukotriene B4
EVLW: Extravascular lung water
IM: Infectious mononucleosis
EV71: Entero virus 71
ARDS: Acute respiratory distress syndrome
APACHE: Acute physiology and chronic health evaluation
SOFA: Sequential organ failure assessment
PLT: Platelet count
PT: Prothrombin time
APTT: Activated partial thromboplastin time
TT: Thrombin time
Fib: Fibrinogen
D-D: D-Dimer
AKI: Acute kidney injury
BUN: Blood urea nitrogen
NR: Not report
